# Urinary metabolites as biomarkers of dietary intake: a systematic review

**DOI:** 10.3389/fnut.2025.1596543

**Published:** 2025-05-15

**Authors:** Mariah Kay Jackson, Bing Wang, Heather Rasmussen, Sathish Kumar Natarajan, Laura D. Bilek, Diane K. Ehlers, Laura Graeff-Armas, Christopher D’Angelo, Teresa Cochran, Kimberly Harp, Corrine Hanson

**Affiliations:** ^1^College of Allied Health Professions, University of Nebraska Medical Center, Omaha, NE, United States; ^2^Department of Food Science and Technology, Institute of Agriculture and Natural Resources, University of Nebraska-Lincoln, Lincoln, NE, United States; ^3^Department of Nutrition & Health Sciences, College of Education and Human Sciences, University of Nebraska-Lincoln, Lincoln, NE, United States; ^4^Department of Quantitative Health Sciences, Mayo Clinic-Arizona, Phoenix, AZ, United States; ^5^Department of Internal Medicine, College of Medicine, University of Nebraska Medicine, Omaha, NE, United States; ^6^Department of Health & Rehabilitation Sciences, College of Allied Health Professions, University of Nebraska Medical Center, Kearney, NE, United States; ^7^Education and Research Services, University of Nebraska Medical Center, Omaha, NE, United States

**Keywords:** urinary metabolites, dietary intake, food groups, dietary biomarkers, polyphenols

## Abstract

**Background:**

Current diet assessment tools, such as food frequency questionnaires, may result in misclassification bias from measurement error and misreporting. These limitations can be mitigated by diet-related biomarkers in urine specimens, an emerging approach to characterize dietary intake.

**Objective:**

We conducted a systematic review to identify urinary biomarkers with utility in accurately assessing dietary intake, including individual foods and food groups.

**Method:**

We retrieved studies from 2000 to 2022 from databases including Embase, CINAHL, Cochrane, and PubMed. Data extraction from included articles was conducted by two independent reviewers for cross validation. Articles identifying urinary biomarkers in relation to food groups/items with adult populations were included and were evaluated for bias using the Joanna Briggs Institute Critical Appraisal.

**Results:**

A total of 65 articles were included and categorized as biomarkers of fruit (*n* = 13), vegetables (*n* = 5), aromatics (*n* = 5), fruits and vegetables (*n* = 3), grains/fiber (*n* = 5), dairy (*n* = 3), soy (*n* = 10), coffee/cocoa/tea (*n* = 9), alcohol (*n* = 6), meat and proteins (*n* = 6), nuts/seeds (*n* = 3), and sugar and sweeteners (*n* = 4). Results expanded the context to which metabolites of foods were compared across similar and dissimilar food groupings. Plant-based foods were often represented by polyphenols, while others were distinguishable by innate food composition, such as sulfurous compounds in cruciferous vegetables or galactose derivatives in dairy.

**Conclusion:**

Current evidence suggests urinary biomarkers may have utility in describing intake of broad food groups, such as citrus fruits, cruciferous vegetables, whole grains, and soy foods, but may lack the ability to clearly distinguish individual foods. These findings indicate the potential of urinary biomarkers to monitor changes in dietary patterns. The improvement of diet assessment methodology is a key step toward strengthening research data validity and accurately measuring outcomes in chronic disease management.

**Systematic review registration:**

https://www.crd.york.ac.uk/PROSPERO/view/CRD42022308255, Prospero CRD42022308255.

## Introduction

Accurate assessment of dietary intake is key in understanding diet-disease relationships. Current diet assessment tools, such as a food frequency questionnaire (FFQ) or a 24-h recall, have been validated and used in producing the preponderance of current evidence of the diet and chronic disease relationship (1, 2). However, a drawback of these tools lies in their self-reported nature, which may result in misclassification bias from measurement error and misreporting. This may ultimately compromise the efficiency and efficacy of dietary interventions, underscoring the need for complementary methodologies for improving assessment accuracy in free-living populations (3, 4). These limitations can be mitigated by diet-related biomarkers. Diet related biomarkers are generally classified as, exposure/recovery biomarkers, which are directly related to dietary intake (e.g., doubly labeled water for energy intake) and outcome/concentration biomarkers, which can be impacted by a person’s individual innate characteristics such as genetics, metabolism or existing health conditions, and thus are an indirect assessment of diet (5–7). New biomarkers are being discovered that have predictive qualities and a more stable dose–response relationship to nutrient intake (5). Blood samples have been used to assess direct circulating levels of nutrients but are often limited by accessibility to lab facilities and invasive collection methods. Urine may be a more accessible and less burdensome biological fluid, with the capability of characterizing dietary intake while having less invasive collection requirements (8, 9).

The 2020–2030 NIH Strategic Plan for Nutrition Research outlines several objectives related to the development of new tools for precision nutrition research. These include assessing the variability of an individual’s diet response through metabolomic profiling/phenotyping, where using biomarkers of dietary exposure can provide more holistic characterizations of diet and mitigate the effects of self-report measurement error (5, 10). Nutrition phenotyping, which is the process of identifying the integrated set of observable measurements that represents overall metabolism of dietary intake (11), can increase the validity and scientific rigor of nutritional status assessment. Consequently, there is a need for the identification of a quantitative measures of dietary intake that can be used to improve dietary assessment. While there have been studies evaluating the use of urinary metabolites, there has been no consensus on the best markers of dietary intake, outside of the widely accepted doubly labeled water for energy intake or urinary nitrogen for protein intake (12). This leaves a clear gap for the assessment of key food groups and dietary patterns. Establishing the efficacy of urinary metabolites for the use of dietary assessment will serve to improve measurement error and bias in collecting dietary data. The objective of this systematic review was to evaluate the urinary biomarkers that can be utilized for accurate assessment of dietary intake, including individual foods and food groups.

## Methods

### Protocol and registration

This protocol is registered at PROSPERO (CRD42022308255) available at https://www.crd.york.ac.uk/prospero/. This protocol was structured according to the items (headings) recommended in the Preferred Reporting Items for Systematic Reviews and Meta-Analyses for systematic review protocols (PRISMA-P) guidelines (13, 14). The PRISMA 2020 checklist is provided in Supplemental materials (15).

### Research question

PICOS (population, intervention, comparison, outcomes, study design) criteria are shown in Table 1 and were used to frame our research question: What are the urinary biomarkers that can be utilized for accurate assessment of dietary intake, including individual foods and food groups?

**Table 1:** PICOS criteria for inclusion.

Population	Adult population with no metabolic diseases (inborn errors of metabolism)
Intervention	Dietary intake, as individual or grouped food items, excluding supplementation and bioavailability studies
Comparison	Other dietary intake interventions
Outcome	Urinary biomarkers related to diet factors
Study design	Interventional Trials, Observational Cohort, Case–Control, Quasi-Experimental, and Cross-sectional

#### Identification of studies

##### Information sources

We conducted the literature search across relevant databases, including PubMed, EMBASE, Cochrane, and CINAHL. The databases were searched from January 2000 through March 2022.

##### Search strategy

A comprehensive list of search terms was compiled, related to the two concepts of the study question: exposure (dietary intake) and outcome (urinary biomarkers). Original search terms were gleaned from 15 relevant benchmark articles and further refined as search terms in Embase, CINAHL, Cochrane, and PubMed. The search terms used included, “biomarker,” “nutritional biomarker,” “dietary biomarker,” “nutrient biomarker,” “food biomarker,” “metabolite,” or “metabolomics,” combined with “diet,” “dietary pattern,” “nutrition phenotyping,” “food,” “food group,” “Western diet,” “Mediterranean diet,” “prudent diet,” or “dietary intake,” as well as, “urine,” “urinary,” or “urinary marker.” Results were limited to English only and human only. All retrieved citations from multiple databases were initially imported into an electronic reference management program (RefWorks). Deduplication was initially performed automatically using an internal function of the program, followed by a manual review for further accuracy. The full search strategy is presented in Supplementary materials.

##### Relevance screening

After the initial search was made, the following inclusion and exclusion criteria were applied. This review included articles evaluating the assessment or identification of urinary biomarkers in relation to food groups or food items with adult populations with no metabolic diseases (inborn errors of metabolism), that were published in English. Only published studies were eligible, including interventional trials, observational cohort, case–control, quasi-experimental, and cross-sectional. Studies were excluded for study populations under the age of 18, non-original research (e.g., review article), or assessment of biomarkers of a non-diet-related nature (e.g., biomarkers of disease-risk/health status, oxidative stress; toxin/pollutant markers). Studies whose objective was methods development of biomarker assessment, validation of a self-report questionnaire, or investigated vitamin/minerals, including supplementation, were excluded.

The study selection process included initial title-and-abstract screening and further full-text assessment, facilitated by Rayyan, using the manual sorting and labeling tools, without the prediction automation feature (16). For both the abstract screening and full-text assessment, reviews were completed by two independent reviewers to ensure articles met all inclusion criteria for data extraction. Abstract screening excluded articles with no relevance to diet and urinary biomarkers, while including those suggestive of meeting inclusion criteria, warranted full-text review. Both reviewers had to deem the article eligible for inclusion for the article to be included. If both reviewers assessed that the article was not eligible for inclusion, then the article was excluded. In the case of discrepancies between reviewers on eligibility status, a third independent reviewer decided on the inclusion or exclusion.

A decision tree was used to guide reviewers through the inclusion and exclusion criteria, based off the following questions, *(1) Correct study population: Is the study population in adults aged 18 or older and does not include those diagnosed with inborn errors of metabolism?; (2) Does the study involve urinary biomarkers/metabolites?; and (3) Does the study relate the urinary biomarkers/metabolites to dietary intake assessment of food/food groups?; or (4) Does the study aim to establish a relationship between urinary metabolites/biomarkers and food/food groups?* Based on these questions, articles were excluded if (1) included children less than 18 years old or included inborn errors of metabolism; (2) examined only non-urine biomarkers (studies that involve both blood/plasma and urinary biomarkers were eligible for inclusion, but only urinary biomarkers were evaluated); (3) examined biomarkers of disease-risk/health status (e.g., cancer markers, cytokines/interleukins, growth factor etc.); biomarkers associated with weight status; biomarkers of oxidative stress; toxin/pollutant markers; (4) objective of assessing supplementation of specific nutrients, non-food derived compounds, or bioavailability; or (5) the study purpose was to use urinary biomarkers to validate a food frequency questionnaire or other reported intake measurement.

##### Data extraction

Data extraction was conducted by two independent reviewers for cross validation. In the case of discrepancies between reviewers on data extracted, a third independent reviewer decided on the correct point for extraction. Data extraction was managed through Research Electronic Data Capture (REDCap) (17, 18), a secure, web-based application designed to support data capture for research studies. For the purposes of this review, REDCap facilitated recording extracted data and managing data organization. A pilot review was conducted by all reviewers on the first ten manuscripts to ensure clarity of the extraction process and that the correct extraction points have been identified. Data extracted were as follows: country of study; study design; author information; sample size; mean age; urine collection method (24-h collection, spot urine collection, multi-spot collection, other/unspecified); dietary assessment method (provided food/controlled feeding trial vs. self-reported intake); urinary biomarkers tested; urinary biomarkers with significant relationships to food/nutrients; and major study limitations.

##### Quality assessment

The Joanna Briggs Institute (JBI) Critical Appraisal Tools were used to assess multiple study types (e.g., randomized controlled trial, observational study) to assess the methodological quality of a study and to determine the extent to which a study has addressed the possibility of bias in its design, conduct and analysis (19). A JBI assessment was made on an included article twice, by two independent reviewers and disagreement on inclusion or exclusion due to unacceptable bias (determined by the respective JBI tool scoring criteria) was reconciled by consulting a third senior investigator of this study. Studies not meeting acceptable JBI criteria were excluded from analysis.

##### Data synthesis

Extracted article data was sorted into the following food groupings for synthesis: biomarkers of fruit, vegetables, fruits and vegetables combined, aromatics, grains/fiber, nuts/seeds/oils, sugar/sweeteners, coffee/cocoa/tea, alcohol, dairy, soy, and meat/proteins. As the level of detail presented in the article allowed, articles were additionally sorted into subgroups in order to describe similar food items, including berries, citrus fruit, and cruciferous vegetables.

## Results

### Study selection

**Figure 1:**
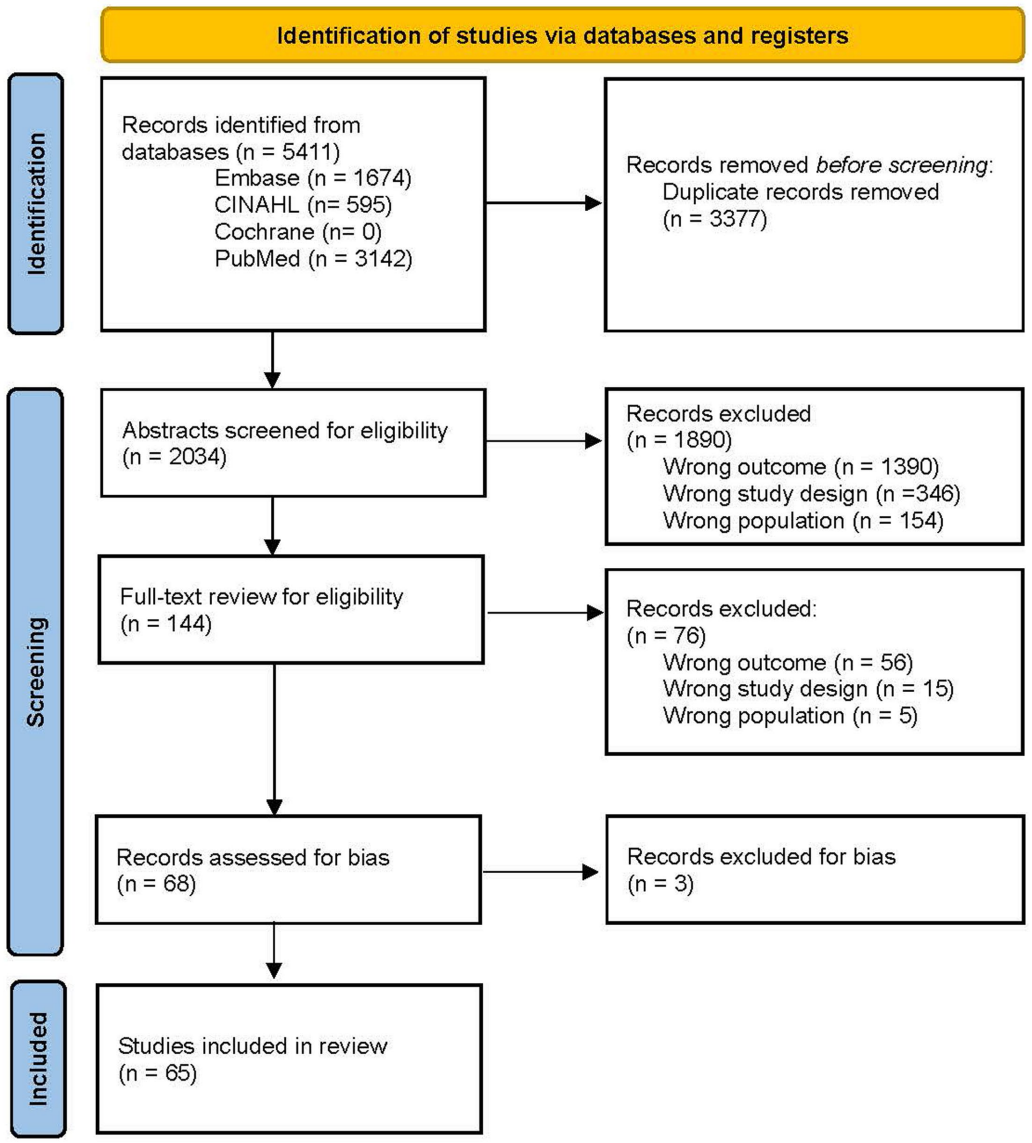
PRISMA 2020 flow diagram for systematic reviews. Adapted from Page et al. (15), licensed under CC BY 4.0.

The complete description of how articles were included and excluded is detailed in Figure 1. In short, the literature search retrieved 5,411 records, where 3,377 duplicates were removed prior to screening, leaving 2,034 abstracts screened for inclusion. Of these, 1,890 records were excluded, leaving 144 records for full text assessment. From the full text review, 68 records underwent bias assessment, where three were excluded for high-risk bias. Therefore, 65 articles were included in the present review.

### Study characteristics

Of the 65 articles identified, there were 39 diet intervention studies, 10 quasi-experimental design studies, and 16 cross-sectional analyses. Publications represented 19 countries, including Australia (1), Austria (1), Canada (1), China (1), Denmark (4), Finland (5), France (1), Germany (6), Ireland (1), Italy (2), Japan (1), Macedonia (1), Malaysia (1), Netherlands (3), Spain (11), Sweden (1), Switzerland (1), United Kingdom (10), and United States (13). The median sample size was 20 participants, where 71% (*n* = 46) of studies had less than 50 participants, 23% (*n* = 15) of studies between 50 and 500 participants, and 6% (*n* = 4) studies over 500 people. The dietary factors investigated were categorized as biomarkers of fruit (*n* = 13), vegetables (*n* = 5), fruits and vegetables combined (*n* = 3), aromatics (*n* = 4), grains and fiber (*n* = 5), nuts/seeds/oils (*n* = 4), sugar and sweeteners (*n* = 4), coffee/cocoa/tea (*n* = 10), alcohol (*n* = 6), dairy (*n* = 3), soy/isoflavones (*n* = 10), and meat and proteins (*n* = 6). Of the 65 studies, the majority used 24-h urine collection methods (*n* = 35), followed by multi-spot collection (*n* = 20), morning spot urine collection (*n* = 6), and other/unspecified (*n* = 4). All cross-sectional studies used either food records (*n* = 7), FFQs (*n* = 6) or 24-h recalls (*n* = 3), while all the interventional/quasi-experimental studies provided food with some degree of control (*n* = 49). Table 2 summarizes the study characteristics.

**Table 2:** Study characteristics of included records (*N* = 65).

Author	Year	Study design	*N*	Country	Food category	Metabolomics methods	Urine collection methods	Diet collection methods
Altorf-van der Kuil	2013	Intervention Study	30	Netherlands	Meat/Protein	Targeted	24-h collection	Provided food/controlled feeding trial
Anesi	2019	Quasi-Experimental	11	Germany	Fruit	Targeted; Untargeted	Multi-spot collection	Provided food/controlled feeding trial
Arai	2000	Cross-sectional	106	Japan	Soy	Targeted	24-h collection	Food records/diary
Atkinson	2002	Cross-sectional	363	USA	Soy	Targeted	24-h collection	Food frequency questionnaire
Aubertin-Leheudre	2008	Cross-sectional	56	Finland	Grains/Fiber	Targeted	24-h collection	Food records/diary
Bresciani	2020	Intervention Study	21	Italy	Cocoa/Coffee/Tea	Targeted	Multi-spot collection	Provided food/controlled feeding trial
Cho	2016	Intervention Study	40	USA	Meat/Protein	Targeted	Multi-spot collection	Provided food/controlled feeding trial
Cross	2011	Intervention Study	17	USA	Meat/Protein	Targeted	24-h collection	Provided food/controlled feeding trial
Cuparencu	2019	Intervention Study	12	Denmark	Meat/Protein	Untargeted	Multi-spot collection	Provided food/controlled feeding trial
Cupareneu	2016	Intervention Study	18	Denmark	Fruit	Untargeted	Multi-spot collection	Provided food/controlled feeding trial
Daykin	2005	Intervention Study	3	Netherlands	Cocoa/Coffee/Tea	Untargeted	24-h collection	Provided food/controlled feeding trial
Edmands	2011	Intervention Study	12	UK	Vegetable	Untargeted	Multi-spot collection	Provided food/controlled feeding trial
Erlund	2001	Quasi-Experimental	13	Finland	Fruit	Targeted	24-h collection	Provided food/controlled feeding trial
Franke	2006	Intervention Study	20	USA	Soy	Targeted	Morning spot collection	Provided food/controlled feeding trial
Frankenfeld	2011	Cross-sectional	3,115	USA	Soy	Targeted	Other/Unspecified	24-h recall
Fraser	2010	Cross-sectional	26	USA	Soy	Targeted	Other/Unspecified	24-h recall
Freedman	2022	Cross-sectional	126	UK, USA	Sugar	Targeted	24-h collection	Food records/diary
Garcia-Aloy	2015	Cross-sectional	155	Spain	Grains/Fiber	Untargeted	Morning spot collection	Food frequency questionnaire
Garg	2016	Intervention Study	14	UK	Grains/Fiber	Untargeted	Multi-spot collection	Provided food/controlled feeding trial
Grace	2004	Cross-sectional	333	UK	Soy	Targeted	Other/Unspecified	Food records/diary
Grainger	2019	Quasi-Experimental	55	USA	Soy	Targeted	24-h collection	Provided food/controlled feeding trial
Haron	2011	Intervention Study	20	Malaysia	Soy	Targeted	Multi-spot collection	Provided food/controlled feeding trial
Harsha	2018	Intervention Study	11	Ireland	Vegetable	Untargeted	Morning spot collection	Provided food/controlled feeding trial
Heinonen	2003	Intervention Study	6	Finland	Soy	Targeted	24-h collection	Provided food/controlled feeding trial
Helander	2005	Quasi-Experimental	9	Sweden	Alcohol	Targeted	Multi-spot collection	Provided food/controlled feeding trial
Hodgson	2004	Cross-sectional	455	Australia	Cocoa/Coffee/Tea	Targeted	24-h collection	24-h recall
Hollands	2008	Quasi-Experimental	10	UK	Fruit	Targeted	Multi-spot collection	Provided food/controlled feeding trial
Hong	2004	Intervention Study	6	USA	Aromatics	Untargeted	Multi-spot collection	Provided food/controlled feeding trial
Hutchins	2000	Intervention Study	34	USA	Nut/Seed/Oil	Targeted	24-h collection	Provided food/controlled feeding trial
Ito	2005	Intervention Study	18	France	Cocoa/Coffee/Tea; Fruit	Targeted	24-h collection	Provided food/controlled feeding trial
Krogholm	2004	Intervention Study	12	Denmark	Fruit;Vegetable	Targeted	24-h collection	Provided food/controlled feeding trial
Kruger	2017	Cross-sectional	297	Germany	Meat/Protein	Targeted	24-h collection	Food records/diary
Lang	2011	Intervention Study	9	Germany	Cocoa/Coffee/Tea	Targeted	Morning spot collection	Provided food/controlled feeding trial
Li	2021	Cross-sectional	246	Netherlands and Switzerland	Dairy	Targeted; Untargeted	24-h collection	Food frequency questionnaire
Lloyd	2011	Cross-sectional	23	UK	Fruit	Untargeted	Multi-spot collection	Food frequency questionnaire
Logue	2017	Intervention Study	12	Italy	Sugar	Targeted	24-h collection	Provided food/controlled feeding trial
Miró-Casas	2003	Intervention Study	7	Spain	Nut/Seed/Oil	Targeted	24-h collection	Provided food/controlled feeding trial
Mullen	2006	Intervention Study	6	UK	Aromatics	Targeted	Multi-spot collection	Provided food/controlled feeding trial
Nielsen	2002	Intervention Study	94	Finland	Fruit;Vegetable	Targeted	24-h collection	Provided food/controlled feeding trial
Pimentel	2020	Intervention Study	11	Switzerland	Dairy	Untargeted	Multi-spot collection	Provided food/controlled feeding trial
Rechner	2002	Quasi-Experimental	10	UK	Fruit	Targeted	24-h collection	Provided food/controlled feeding trial
Rechner	2001	Quasi-Experimental	5	UK	Cocoa/Coffee/Tea	Targeted	24-h collection	Provided food/controlled feeding trial
Roura	2008	Intervention Study	21	Spain	Cocoa/Coffee/Tea	Targeted	Multi-spot collection	Provided food/controlled feeding trial
Saenger	2017	Quasi-Experimental	30	Germany	Fruit	Targeted	Multi-spot collection	Provided food/controlled feeding trial
Saenger	2021	Quasi-Experimental	32	Germany	Fruit	Targeted	Multi-spot collection	Provided food/controlled feeding trial
Scheffler	2016	Intervention Study	12	Germany	Aromatics	Targeted	Multi-spot collection	Provided food/controlled feeding trial
Söderholm	2011	Quasi-Experimental	15	Finland	Grains/Fiber	Targeted	Multi-spot collection	Provided food/controlled feeding trial
Song	2013	Intervention Study	82	USA	Sugar	Targeted	24-h collection	Provided food/controlled feeding trial
Stanoeva	2013	Intervention Study	10	Macedonia	Cocoa/Coffee/Tea	Targeted	24-h collection	Provided food/controlled feeding trial
Sun	2020	Intervention Study	6	USA	Vegetable	Targeted; Untargeted	Multi-spot collection	Provided food/controlled feeding trial
Tasevska	2004	Intervention Study	12	UK	Sugar	Targeted	24-h collection	Provided food/controlled feeding trial
Tomás-Navarro	2021	Intervention Study	18	Spain	Fruit	Untargeted	24-h collection	Provided food/controlled feeding trial
Toren	2005	Cross-sectional	68	Canada	Dairy	Targeted	24-h collection	Food records/diary
Toromanović	2008	Intervention Study	10	Austria	Fruit	Targeted	24-h collection	Provided food/controlled feeding trial
Tulipani	2011	Intervention Study	42	Spain	Nut/Seed/Oil	Untargeted	24-h collection	Provided food/controlled feeding trial
Tulipani	2012	Intervention Study	41	Spain	Nut/Seed/Oil	Targeted	24-h collection	Provided food/controlled feeding trial
Ulaszewska	2016	Intervention Study	126	USA	Fruit;Vegetable	Untargeted	24-h collection	Provided food/controlled feeding trial
Upi-Sarda	2009	Intervention Study	42	Spain	Cocoa/Coffee/Tea	Targeted	24-h collection	Provided food/controlled feeding trial
Urpi-Sarda	2015	Intervention Study	36	Spain	Alcohol	Targeted	24-h collection	Provided food/controlled feeding trial
Vazquez-Fresno	2015	Intervention Study	261	Spain	Alcohol	Untargeted	24-h collection	Provided food/controlled feeding trial
Wang	2021	Cross-sectional	648	USA	Fruit; Vegetable; Aromatics; Grains/Fiber; Cocoa/Coffee/Tea; Alcohol; Meat/Protein	Untargeted	24-h collection	Food records/diary
Wu	2012	Cross-sectional	2,165	China	Soy	Targeted	Other/Unspecified	Food frequency questionnaire
Xi	2022	Intervention Study	19	Denmark	Vegetable	Untargeted	24-h collection	Provided food/controlled feeding trial
Zamora-Ros	2009	Cross-sectional	1,000	Spain	Alcohol	Targeted	Morning spot collection	Food frequency questionnaire
Zamora-Ros	2006	Intervention Study	20	Spain	Alcohol	Targeted	Morning spot collection	Provided food/controlled feeding trial

### Fruit, vegetables, and aromatics

**Figure 2:**
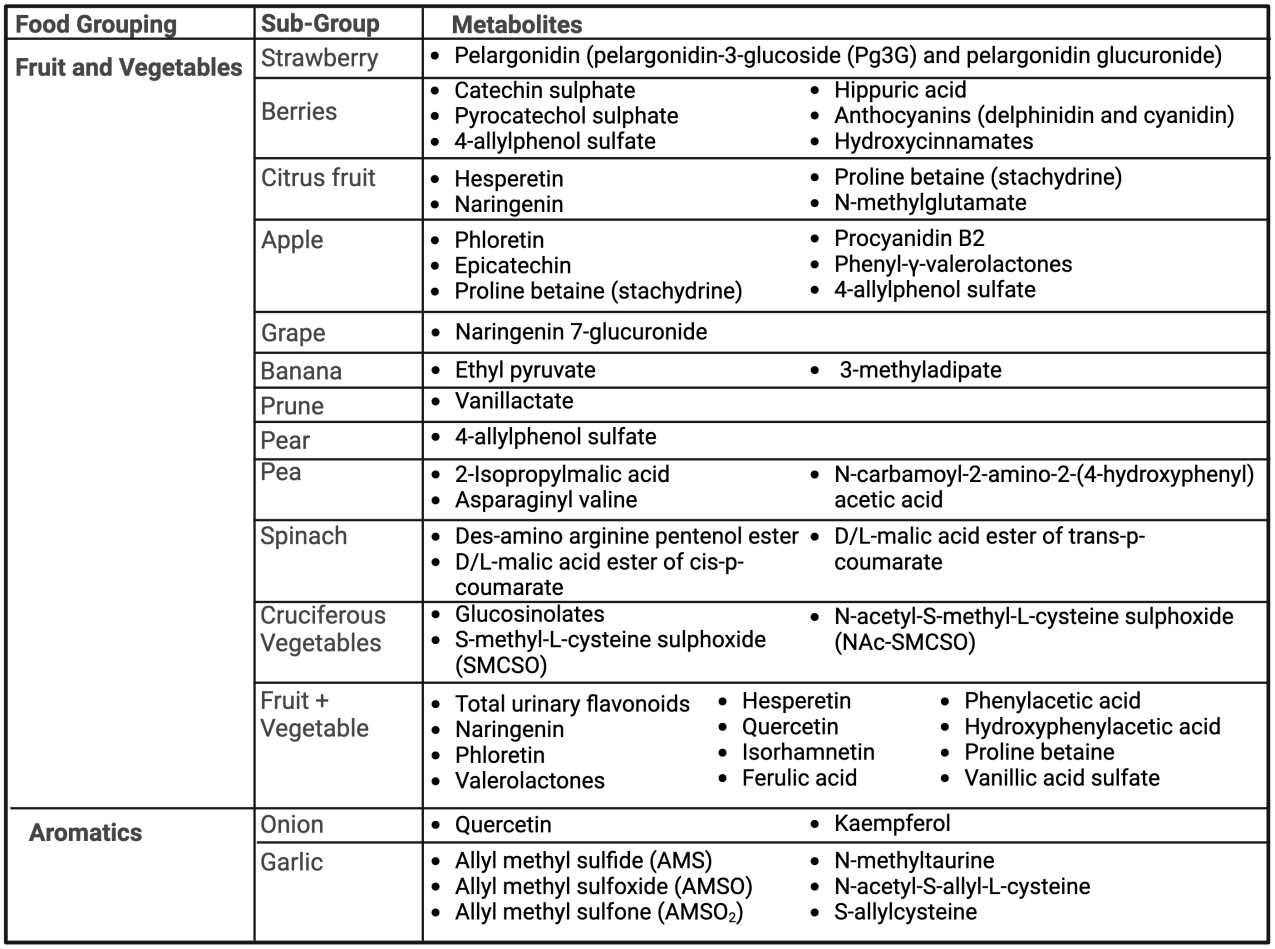
Summary of urinary metabolites as biomarkers of fruits, vegetables, and aromatics. Created with BioRender.com. Jackson, M. (2025) https://BioRender.com/w44s987.

Figure 2 summarizes the urinary metabolites proposed as biomarkers of dietary intake across studies for fruit, vegetables and aromatics.

#### Fruit

Several studies included berries (20–25). Pelargonidin, an anthocyanidin, and its derived forms were found by two studies as potential biomarkers of strawberries, including pelargonidin-3-glucoside (Pg3G) and pelargonidin glucuronide (20, 21). Hollands et al. (21) found a significant linear dose–response relationship of the consumption of strawberries to urinary excretion of strawberry anthocyanins, where pelargonidin-3-glucoside (Pg3G) made up 93% of total anthocyanins found in strawberries. Berry consumption biomarkers, of assorted varieties, include many polyphenols, including catechin sulphate (20, 25), pyrocatechol sulphate (20), 4-allylphenol sulfate (25), as well as the related polyphenol metabolite, hippuric acid (20, 22). Of these, Toromanovic et al. found consumption of blueberries and cherries produced the highest urinary excretion of hippuric acid, which was significantly higher as compared to a mixed food control (22). Rechner et al. (24) investigated ingestion of black currant juice, which is rich in anthocyanins, and urinary metabolites as biomarkers. After following a polyphenol-free diet for 2 days, four anthocyanins related to delphinidin and cyanidin, as well as hydroxycinnamates, flavonols, and urinary hippuric acid were detected.

Citrus fruits were also commonly examined, including orange and grapefruit (23, 25–30). Proline betaine (also called stachydrine) (25, 27–30), hesperetin (23, 26, 28, 29), naringenin (23, 26, 28, 29), and N-methylglutamate (25) were found to be urinary biomarkers of orange/orange juice intake. Similarly, grapefruit intake was significantly associated with urinary proline betaine (27), naringenin (23) and, N-methylglutamate (25). Only proline betaine was able to distinguish between consumption amounts and be detectable for at least 72 h, whereas hesperetin, and naringenin were determined to be only short-term qualitative biomarkers of orange juice (28). Short term urinary excretion of hesperetin and naringenin was also found by Elrund et al. after consumption of 8 mL/kg of orange juice (26). Furthermore, proline betaine was found to be a distinguishable urinary biomarker from both reported habitual intake and acute provision of citrus foods (orange and grapefruit) (27) and was dose-dependent (30). Tomás-Navarro et al. (29) described distinguishable biomarkers that exist between orange juice processing methods, including higher levels of sinapic acid derivatives in processed juices, likely from higher exposure to peel oils. However, high levels of hesperetin and naringenin were present across all fresh and processed orange juices (29).

Two studies investigated urinary biomarkers of apple consumption. Saenger et al. examined the urinary biomarkers associated with low (1 apple; ~200 g), medium (2 apples; ~400 g) or high (4 apples; ~800 g) apple consumption following a three-day wash out period of no apple product intake (31). Levels of phloretin, epicatechin, and procyanidin B2 significantly increased following apple consumption and could distinguish between high and low apple intake but could only be detected up to 12–24 h (31). As apples are a source of flavan-3-ols, Anesi et al. (32) investigated the use of phenyl-*γ*-valerolactones as a biomarker of flavan-3-ols from apples. Phenyl-γ-valerolactones were able to be detected after apple consumption, most notably between 6 and 12 h post-intake. Like oranges, proline betaine was also a predictive metabolite for apple intake (25). Wang et al. established predictive metabolites for 79 food groups/individual food items based off of FFQs and 24 h recalls. For fruits, naringenin 7-glucuronide was a predictive biomarker for grape intake, vanillactate for prunes, ethyl pyruvate and 3-methyladipate for banana, and 4-allylphenol sulfate for apples or pears (25).

#### Vegetables

Various vegetables including peas (33), spinach (34), and several cruciferous vegetables, as a group (25) and individually, including kale (35), daikon radish (35), broccoli (36) and Brussels sprouts (36) were examined.

Biomarkers of pea intake were assessed through a dose–response randomized cross-over trial comparing 4 days of low (40 g), medium (75 g) and high (165 g) pea intake to a couscous control meal (33). Additionally, results were verified in an independent confirmation study of a pea-protein burger compared to meat. From the dose–response trial, 2-Isopropylmalic acid, asparaginyl valine and N-carbamoyl-2-amino-2-(4-hydroxyphenyl) acetic acid were significantly different after pea consumption and increased in a dose–response manner (33). These three biomarkers were also confirmed after ingestion of a pea-protein burger compared to a meat-based burger (33).

Both whole leaf and minced spinach consumption was investigated through a randomized cross-over design (34). After ingesting 178 g of whole spinach or 200 g of minced spinach, three biomarkers were identified as increasing post-consumption: des-amino arginine pentenol ester, D/L-malic acid ester of cis-p-coumarate, and D/L-malic acid ester of trans-p-coumarate. Results were similar for both spinach preparations (34).

Sun et al. examined urinary biomarkers of kale and daikon radish as representative *Brassica* cruciferous vegetables (35). Six participants were provided 250 g of steamed baby kale and 25 g of raw daikon radish and 24-h urine was collected. Post consumption, 18 metabolites were identified, including four phenolic compounds and 14 glucosinolates. Kale exhibited higher phenolic compound levels than daikon radish and daikon radish showed higher total glucosinolate levels (35). Excretion rates often peaked within 6 h and thus may be reflective of short-term intake versus habitual. Edmands et al. investigated two other cruciferous vegetables, broccoli and Brussels sprouts, with urine collection over 48 h (36). Twelve participants participated in a controlled diet intervention study, with phases of high (250 g broccoli and Brussels sprouts) and low cruciferous vegetable intake (excluded cruciferous vegetables and *alliums*). From the intervention, S-methyl-L-cysteine sulphoxide (SMCSO) and N-acetyl-S-methyl-L-cysteine sulphoxide (NAc-SMCSO) were identified as stable urinary biomarkers of cruciferous vegetables (36). Wang et al. (25) similarly confirmed S-methylcysteine sulfoxide as a predictive metabolite for cruciferous vegetables.

#### Fruit and vegetable intake

The use of flavonoids as a biomarker of fruit and vegetable intake were investigated by three studies (30, 37, 38). Neilsen et al. and Krogholm et al. both conducted controlled intake trials, providing diets of low and high fruit and vegetable intake (37, 38). Similar findings from these two studies demonstrated total urinary flavonoids and kaempferol have dose-dependent relationships with fruit and vegetable intake (37, 38). However, while Nielsen et al. (38) additionally found significant differences between highest and lowest quartiles of diet intake and urinary excretion of naringenin and phloretin, Krogholm et al. (37) did not find phloretin were to be significantly associated with the exposure of high vs. low fruit and vegetable intake. Hesperetin (38), quercetin (37), and isorhamnetin (37) may additionally show dose-dependent relationships with fruit and vegetable intake, while two valerolactones and six benzoic acid derivatives, including ferulic acid, vanillic acid sulfate, phenylacetic acid, hydroxyphenylacetic acid, and proline-betaine were found to be markers of long-term exposure to high flavonoid intake from fruits and vegetables (30).

#### Aromatics

Four studies investigated aromatics of onion (25, 39, 40) and garlic (25, 41). Quercetin metabolites were significantly associated with onion intake (39, 40). Additionally, four isomers of kaempferol monoglucuronides were found, with kaempferol being the second most abundant flavonoid after quercetin in onions (39). Wang et al. (25) also identified N-methyltaurine, 2,3-dimethylsuccinate, N-acetylalliin as predictive metabolites of onion intake.

Scheffler et al. recruited twelve volunteers to provide 24-h urine samples after the ingestion of 3 g (1–2 cloves) of raw garlic, where one volunteer ate 30 g (approximately one bulb) of raw garlic (41). After garlic consumption, three primary metabolites were found: allyl methyl sulfide (AMS), allyl methyl sulfoxide (AMSO) and allyl methyl sulfone (AMSO_2_). These metabolites may be reflective of short-term intake, with decreasing concentrations generally after 3–6 h post-consumption (41). Wang et al. (25) also identified N-methyltaurine, N-acetyl-S-allyl-L-cysteine, and S-allylcysteine as predictive metabolites of garlic intake.

### Grains and fiber

**Figure 3:**
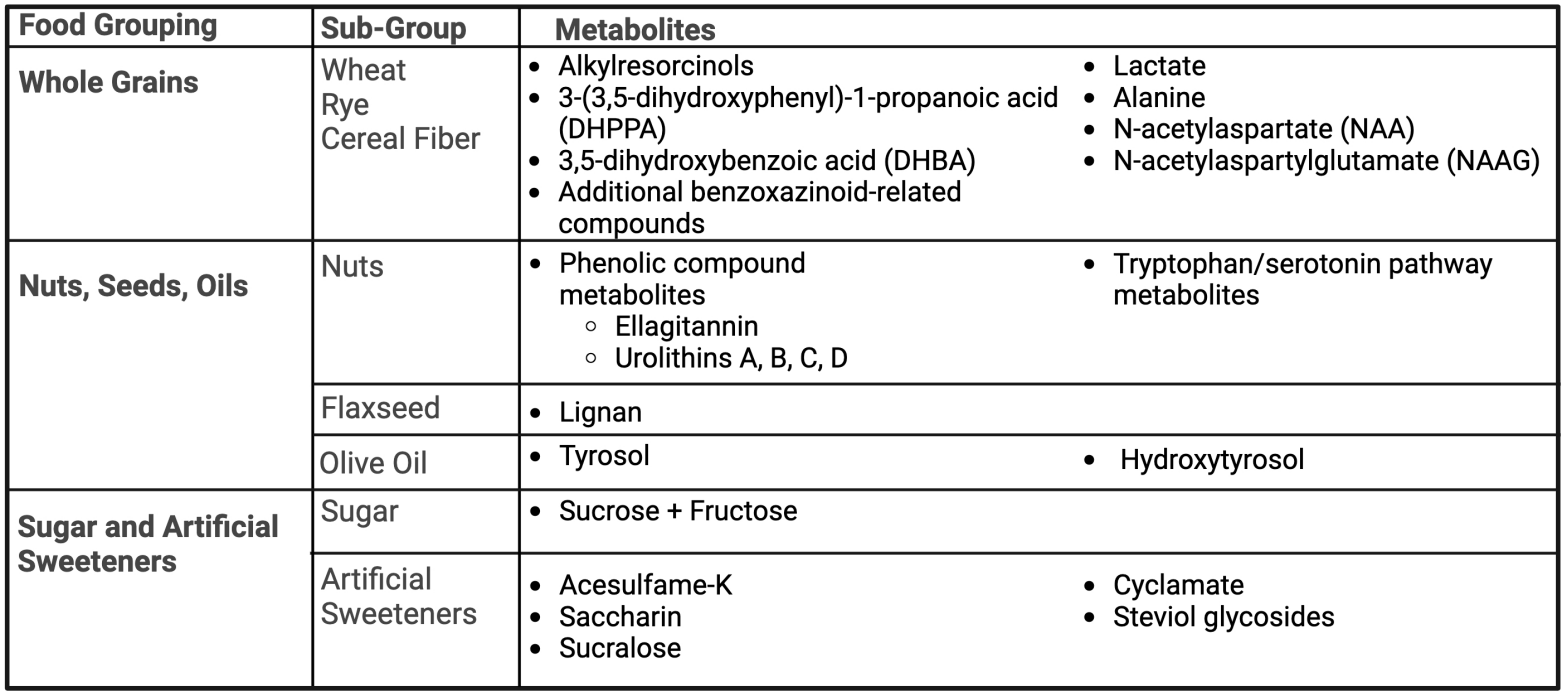
Summary of urinary metabolites as biomarkers of whole grains, nuts, seeds, oils, and sugar. Created with BioRender.com. Jackson, M. (2025) https://BioRender.com/t08g470.

A summary of the metabolites for whole grains and fiber is presented in Figure 3. Alkylresorcinols, and their metabolites, 3-(3,5-dihydroxyphenyl)-1-propanoic acid (DHPPA) and 3,5-dihydroxybenzoic acid (DHBA), found in wheat and rye products, have been identified as biomarkers of whole grain intake by three studies (42–44). Söderholm et al. (44) investigated the consumption of 198 g of rye bread, containing 100 mg of alkylresorcinols, in 15 healthy volunteers and subsequent urinary biomarkers after 25 h of urine collection. Metabolites of alkylresorcinols, DHPPA and DHBA, were present after consumption of rye bread. Maximum excretion of these metabolites was approximately 5–6 h, with low levels still detectable at 25 h (44). Aubertin-Leheudre et al. also investigated the metabolites of alkylresorcinols as biomarkers of whole grain wheat and rye cereal intake in 56 Finnish women (42). Three-days of diet records and urine collection were collected and analyzed for urinary concentrations of DHPPA and DHBA in relation to fiber and fiber-rich food intake. Total fiber intake was correlated with urinary DHBA, and cereal fiber intake was correlated with both DHBA and DHPPA, including adjustment for age and BMI. Furthermore, DHPPA was an independent predictor of cereal fiber intake after adjustment (42). Garcia-Aloy et al. (43) was able to distinguish the presence of several metabolites, including alkylresorcinol derivatives, by comparing non-bread consumers (*n* = 56), white bread consumers (*n* = 48), and whole-grain bread consumers (*n* = 51) in a free-living sub-population enrolled in the PREDIMED Study. Stratified by typical consumption, as reported by FFQ, subjects provided a fasting morning spot urine sample. Alkylresorcinol metabolites DHPPA glucuronide and 5-(3,5-dihydroxyphenyl) pentanoic acid (DHPPTA) sulphate were found to be significantly higher in whole-grain bread consumers compared to white bread consumers and non-bread consumers. Additionally, microbial-derived compounds such as hydroxybenzoic acid glucuronide, as well as benzoxazinoid-related compounds (N-(2-hydroxyphenyl) acetamide (HPAA) glucuronide; 2-hydroxy-7-methoxy-2H-1,4-benzoxazin-3-one (HMBOA)) were excreted in higher amounts by both types of bread consumers compared to non-bread consumers (43). Similarly, DHBA and 2,6-dihydroxybenzoic acid were also able to predict whole grain intake (25). Lastly, Garg et al. investigated the urinary metabolites post-consumption of wheat bran or the isolated component of bran, aleurone (45). Fourteen people were included in this randomized cross-over study, providing either 50 g of minimally processed wheat bran, 50 g of minimally processed wheat aleurone, or a control meal, separated by week-long washout periods. Urine was collected prior to consumption, and again analyzed at hours 1 and 2 post-consumption. There were distinguishable higher levels of lactate, alanine, N-acetylaspartate (NAA) and N-acetylaspartylglutamate (NAAG) after bran and aleurone consumption, but not with the control. Metabolites were not distinguishable between bran and aleurone (45).

### Nuts, seeds and oils

Nuts, seeds and oils are high in polyphenols therefore, these metabolites were tested as biomarkers across several studies. Tulipani et al. designed a randomized controlled trial of a control and nut intervention group (30 g/day of raw unpeeled mixed nuts: 15 g of walnuts, 7.5 g of almonds, and 7.5 g of hazelnuts) (46, 47). Three classes of biomarkers candidates were identified, including fatty acid metabolites, phenolic compound metabolites, and tryptophan/serotonin pathway metabolites (46). Upon further analysis, the phenolic compounds group, specifically the ellagitannin-derived urolithins A and B, were found to significantly increase after nut consumption and urolithins C, and D were also detected (47).

Flaxseed is a lignan-rich food, therefore Hutchins et al. investigated the relationship between dietary intake of flaxseed and urinary metabolites of lignan (48). Healthy post-menopausal women participated in a randomized cross-over study testing 5 and 10 g of ground flaxseed versus a control period, collecting 24-h urine. Both doses increasing lignan metabolite excretion, and was able to demonstrate a dose–response relationship (48).

Miro-Casas et al. examined polyphenols of tyrosol and hydroxytyrosol as urinary biomarkers of olive oil intake (49). Olive oil was given at doses of 50 mL for day 1 and 25 mL for 1 week to test single versus sustained intake. Single and sustained intake of olive oil increased excretion of tyrosol and hydroxytyrosol, with authors recommending tyrosol as a better biomarker due to the dose-effect relationship (49). A summary of the metabolites for nuts, seeds, and oils is presented in Figure 3.

### Sugar and artificial sweeteners

Urinary sugar biomarkers, as well as artificial sweeteners, have been explored within US and UK populations. One of the first sugar biomarker studies, by Tasveka et al., (50) conducted a randomized controlled cross-over trial for 30 days, examining the relationship between intake of sugar at three levels (9.5, 21.8 and 40.2% of energy intake) and urinary sucrose and fructose, as well as a habitual diet verification study. Sugar intake was highly correlated with urinary the combination of urinary sucrose and fructose in both the dose response study and the habitual intake study, with correlation coefficients greater than 0.84 in both analyses. The sum of urinary fructose and sucrose explained 74% of regression model variability from the dose response study and 72% in the habitual intake study. Similarly, Song et al. (51) utilized the CARB (Carbohydrates and Related Biomarkers) randomized cross-over study of 53 participants after following both high and low glycemic-index diets, measuring sucrose and fructose in 24-h urine. While urinary sucrose and fructose were associated with total sugar consumption, only 44.3% of urinary fructose variation and 41.7% of both urinary sucrose and fructose variation were explained by the models adjusted for age, gender, and percent body fat. Freedman et al. utilized an UK-based feeding trial and an Arizona, US-based feeding trial to examine sugar intake and urinary sugar (fructose and sucrose). This study was able to confirm the relationship between urinary sugar and total sugar intake across different populations, generating similar predictive models in both cohorts (52). Urinary biomarkers of artificial sugar intake, including acesulfame-K, saccharin, sucralose, cyclamate, and steviol glycosides, was assessed by Logue et al. via a randomized cross-over dose–response study in 21 adults. Mean urinary concentrations of each artificial sweetener were significantly correlated to provided intake amounts, with correlation coefficients at 0.89 or greater. Regression modeling of the 24-h urinary excretions revealed that a high percent of the variations were explained for acesulfame-K (99%), saccharin (87%), cyclamate (91%) and steviol glycosides (75%), while only accounting for 35% of the variability of sucralose (53).

### Cocoa, coffee, tea

**Figure 4:**
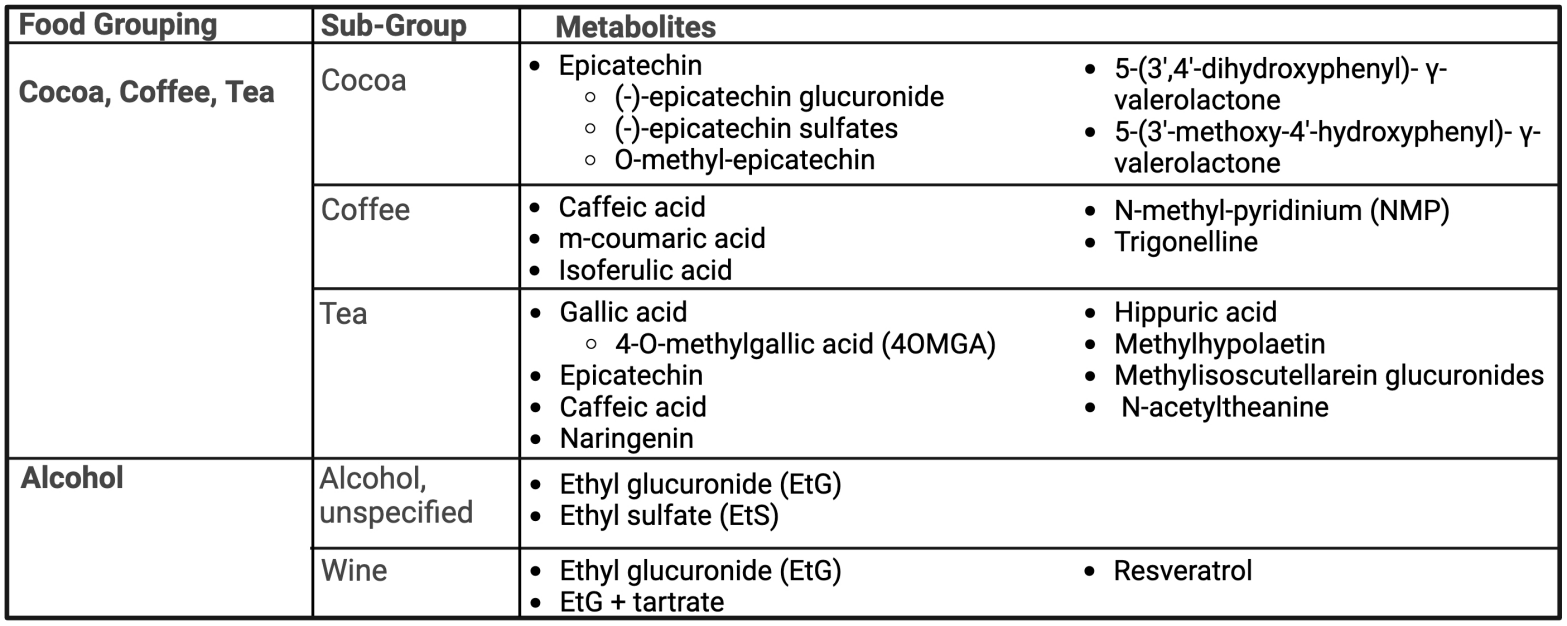
Summary of urinary metabolites as biomarkers of cocoa, coffee, tea, and alcohol. Created with BioRender.com. Jackson, M. (2025) https://BioRender.com/e82j056.

Cocoa, coffee, and tea are high in polyphenols and flavonoids, which was the similar aim among many studies, however specific findings varied greatly, as summarized in Figure 4.

#### Cocoa

Cocoa is a flavonoid-rich food, with epicatechin identified as a biomarker in three studies (23, 54, 55). Roura et al. (54) noted cocoa is commonly ingested in combination with milk, and thus aimed to study the difference in urinary flavonoid metabolites identifiable after consumption of 40 g of cocoa powder prepared with milk or water, compared to milk-only control. Metabolites captured in both cocoa preparations included an (−)-epicatechin glucuronide and three (−)-epicatechin sulfates, showing no differences in overall excretion between treatments. However, findings may suggest the presence of milk may alter flavonoid metabolism, inducing the excretion of sulfates before glucuronides (54). Long-term exposure to cocoa was examined by Urpi-Sarda et al. (55) through a four-week randomized controlled, cross-over trial. Participants received 40 g of cocoa in milk compared to milk-only control for 4 weeks each, in a random order, and collected 24-h urine. Similar to Roura et al. (55) Urpi-Sarda found the presence of (−)-epicatechin glucuronides and (−)-epicatechin sulphates after consumption of cocoa, as well as O-methyl-epicatechin, 5-(3′,4′-dihydroxyphenyl)- *γ*-valerolactone and 5-(3′-methoxy-4′-hydroxyphenyl)- γ-valerolactone. Finally, Ito et al. (23) also confirmed urinary excretion of epicatechin after consumption of a cocoa.

#### Coffee

Ito et al. (23) demonstrated caffeic acid, as well as its metabolite, m-coumaric acid, to be present in urine after coffee ingestion. Hodgson et al. (56) found isoferulic acid was correlated with usual and current coffee intake. Isoferulic acid was able to predict coffee intake status with 57% specificity and 61% sensitivity (56). Similarly, Rechner et al. (57) in a small study of five healthy males, identified isoferulic acid as a unique biomarker after ingestion of instant coffee. Lang et al. (58) sought to define dietary biomarkers of coffee consumption, distinct from other caffeinated foods. N-methyl-pyridinium (NMP) and trigonelline were found to be suitable markers for coffee intake, with detectable presence up to 48 h (trigonelline) and 72 h (NMP) (58). Bresciani et al. also showed trigonelline and NMP had dose–response excretion curves for coffee intake. The authors also noted potential sex-based differences in absorption of trigonelline and that NMP may be impacted by smoking status (59).

#### Tea

Gallic acid, a phenolic acid, and its derivative, 4-O-methylgallic acid (4OMGA) have been identified as a biomarker of tea intake in three studies (23, 56, 60). In Hodgson et al., even after adjustment for age, gender and study group, 4-O-methylgallic acid (4OMGA) was significantly correlated with usual and current tea intake and 4OMGA was able to predict tea-drinking status with 81% specificity and 82% sensitivity (56). Ito et al. also identified the presence of gallic acid after tea consumption, as well as epicatechin, caffeic acid and naringenin (23). Daykin et al. also investigated tea biomarkers, hypothesizing that as an abundant source of polyphenols, flavonoid metabolites would be able to be identified in urine collection (60). This small study of three volunteers in a 5-day tea consumption trial identified hippuric acid as the main metabolite after the consumption of black tea but was only identifiable after around 10.5 h post-consumption, compared to 1,3-dihydroxyphenyl-2-O-sulfate that was present within 5 h of consumption. Low levels of gallic acid were also identified (60).

Urinary biomarkers of a herbal “mountain tea,” *Sideritis scardica*, used primarily by inhabitants of Balkan and Mediterranean countries was examined (61). Flavonoids were the predominate urinary metabolite group, making up to 94% of the total polyphenolic metabolites detected. Of sixty-three metabolites, isomers of methylhypolaetin and methylisoscutellarein glucuronides were most abundant (61). Wang et al. (25) found N-acetyltheanine to be the most predictive biomarker for total tea intake and specifically green tea and black tea.

### Alcohol

A summary of urinary biomarkers of alcohol intake is presented in Figure 4. While alcohol is predominantly metabolized to acetaldehyde and acetic acid, a small fraction is also converted to ethyl glucuronide (EtG) and ethyl sulfate (EtS). This has prompted researchers to examine the utility of EtG and EtS as sensitive biomarkers of alcohol intake. Across three studies, EtG was a biomarker for alcohol intake (25, 62, 63). Helander and Beck conducted a small study with nine healthy adults who drank alcohol (unspecified) equivalent to either 0.15 g/kg or 0.5 g/kg and collected urine for 24 h (62). Both EtS and EtG were identifiable as early as one-hour post-consumption, where EtS exhibited a longer and dose-dependent elimination half-life and was still detectable at 24 h. Notably, water dilution was accounted for when expressed as a ratio of EtS to urinary creatinine (62). While Vazquez-Fresno et al. (63) also found EtG to be a robust marker of wine intake among a sub-population of the free-living PREDIMED cohort study, a combined model of EtG and tartrate produced an AUC of 90.7%, compared to EtG singularly (AUC 86.3%). EtG and tartrate models may also provide evidence of wine consumption between 24 and 72 h, as concentrations of EtG and tartrate were significantly higher in those reporting drinking wine within the previous 3 days compared to non-drinkers, but this difference was not seen in those who drank wine greater than 3 days prior to the urine sample and non-drinkers (63).

Resveratrol is another highly studied component of wine and shown as a potential biomarker of wine intake in three studies (64–66). Two interventional studies were able to distinguish resveratrol as a biomarker of wine intake, compared to gin as a control ethanol source (64, 65), as well as its continued presence in dealcoholized wine (64). Another factor of consideration is white versus red wine, where resveratrol metabolites significantly increase for both white and red wine drinkers, but red wine produced a higher concentration change of resveratrol over white wine (65). In cross-sectional analyses, those reporting wine consumption had significantly higher levels of resveratrol metabolites than non-drinkers (65, 66). Resveratrol metabolites in urine also showed detectable differences between those reporting drinking one glass of wine per week and three glasses of wine per week, where one glass of wine per week was detectable up until 3 days post-consumption and three glasses of wine were detectable until 5 days post-consumption (66).

### Dairy

**Figure 5:**
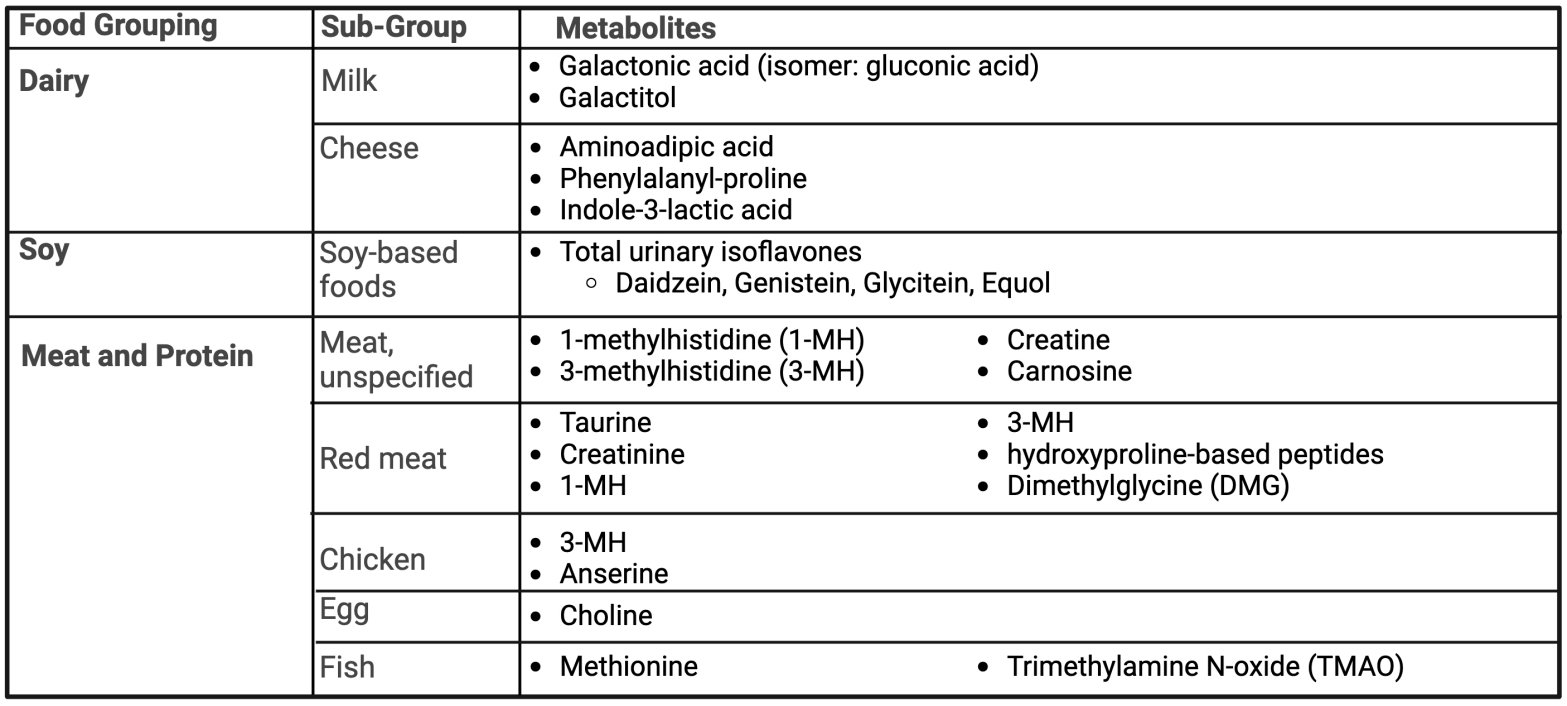
Summary of urinary metabolites as biomarkers of dairy, soy, meat and other protein foods. Created with BioRender.com. Jackson, M. (2025) https://BioRender.com/l61v909.

Products of galactose, a dairy sugar, metabolism have been found to be biomarkers of dairy, including galactonic acid (67) and galactitol (68), summarized in Figure 5. Pimentel et al. utilized the A Healthy Diet for a Healthy Life: Food Biomarkers Alliance (FoodBAll) study to describe potential biomarkers of milk and cheese intake, comparing 600 mL of full-fat milk and 100 g of hard cheese to 600 mL of a soy-based drink with a 24-h urine collection (67). After consumption of milk, urinary blood group H disaccharide (BGH) and galactonic acid and its isomer, gluconic acid were present. Aminoadipic acid, phenylalanyl-proline and indole-3-lactic acid were detected in urine post-cheese consumption; however, aminoadipic acid was not found to be discriminate to cheese, as increased presence was noted with both milk and soy intake (67). Similarly, Li et al. (68) examined intake of milk, cheese, and yogurt in a free-living population in the Netherlands. For milk intake, urinary galactitol was significantly different between quintile 3–5 versus 1–2. Several differences were found based on participant phenotypes, including sex-specific differences in urinary lactose and galactitol in men versus women. Cheese intake had no specific biomarkers for the whole population, but when stratified by sex, urinary indole-3-lactic acid was different across quintile in men, but not women. There were no specific significant markers for yogurt intake (68). Lastly, Toren and Norman investigated the utility of 24-h urinary calcium as a marker for dietary calcium intake. In a population of 68 women, there was no significance between 24-h urinary calcium and dietary calcium, after adjustment (69).

### Soy

Ten studies investigated urinary isoflavones from soy-based foods (Figure 5). There was a consistent significant relationship among various populations and demographics between soy products and urinary isoflavone concentrations. This relationship persisted among Asian cohorts (70, 71) with typical high intake of soy-based foods and Western populations with low reported intake (72–75). Four additional interventional studies (76–79) provided additional evidence of this relationship being present in both men and women. Haron et al. compared urinary isoflavone excretions in post-menopausal women after consumption of tempeh versus milk in a randomized cross-over study. Daidzein and genistein, but not equol, were detected in low amounts after consumption of milk. However, concentrations of daidzein, genistein and equol were up to 13 times higher with tempeh consumption compared to milk, offering a clear distinction (78). Grainger et al. studied 55 men with prostate cancer, prior to prostatectomy, for the relationship of urinary biomarkers after the consumption of zero, one or two 6-oz cans of a tomato-soy drink (77). Urinary isoflavones (daidzein, genistein, glycitein and their derivatives) were only detectable in those consuming the drink, with a significant dose–response relationship.

## Meat and protein sources

Multiple studies covered topics of protein sources, including red meat, fish, eggs, chicken, and pork, where 1-methylhistidine (1-MH) (80, 81), 3-methylhistidine (3-MH) (25, 81, 82) and carnosine (81, 82) were commonly found across studies as biomarkers of meat intake (Figure 5). Additional markers identified urinary choline for egg consumption (83), methionine (83) and Trimethylamine N-oxide (TMAO) (84) for fish consumption, and dimethylglycine (DMG) for beef consumption (83).

Cross et al. utilized 24-h urine samples from two cross-over randomized controlled dietary studies, following different doses of red meat and a high-protein vegetarian diet (80). Urinary output of taurine, creatinine, 1-MH and 3-MH were significantly higher after consumption of a high-red meat diet vs. low red meat or vegetarian diet but only 1-MH and 3-MH increased in a significant dose-dependent manner. Altorf-van der Kuil et al. looked at metabolite profiles for meat, dairy and grain-based protein intake (81). A prediction model using urinary carnosine, 1-MH and 3-MH accounted for 98% of the variability of intake of meat-based protein, but were unable to distinguish between dairy-based protein and grain-based protein (81). Cuparencu et al. (82) designed a randomized cross-over meal study to examine biomarkers for meat consumption, testing 48-h urine after intake of chicken, pork, beef against a control of egg whites and peas. Of the metabolites, creatine and carnosine were confirmed as biomarkers of general meat intake by an independent confirmation study comparing beef intake to a vegetarian meal. Five hydroxyproline-based peptides were confirmed as markers of red meat intake and anserine and 3-MH were detected as biomarkers of chicken intake. However, the study concluded that prediction models were strongest when combining two or more markers (82).

## Discussion

This systematic review demonstrated current evidence for potential urinary biomarkers of food and food group intake. Here, we described a wide array of investigated biomarkers, across food groupings, where some food groups showed multiple potential biomarkers, while others have a narrower consensus. We were able to expand the context (85) to which metabolites of foods of interest were examined and compare them across similar and dissimilar food groupings.

Seemingly, the scope of food studies that investigate one food, while successfully identifying key metabolites of interest, limits the ability to understand shared metabolites across food groupings. In isolation, this may lead to a false conclusion, weakening the ability to correctly distinguish unique biomarkers. For example, kaempferol and quercetin were found to be the two most abundant flavonoids in onions (39), but in separate studies, these also were found to have dose-dependent relationships with fruit and vegetable intake (37, 38). Quercetin and kaempferol are specifically flavonols, a sub-class of flavonoids, which belong to the overarching group of polyphenols (86). Therefore, quercetin and kaempferol, while abundant in onions, may be too ubiquitous biomarker to solely measure onion intake, and rather suggestive of a diet comprised of flavonoid-rich foods, like fruits, vegetables, and aromatics. This has been similarly demonstrated by Yang et al. where those consuming a polyphenol-rich diet (fruits, vegetables, coffee and tea) had significantly higher urinary levels of several polyphenols, including kaempferol and quercetin (87).

**Figure 6:**
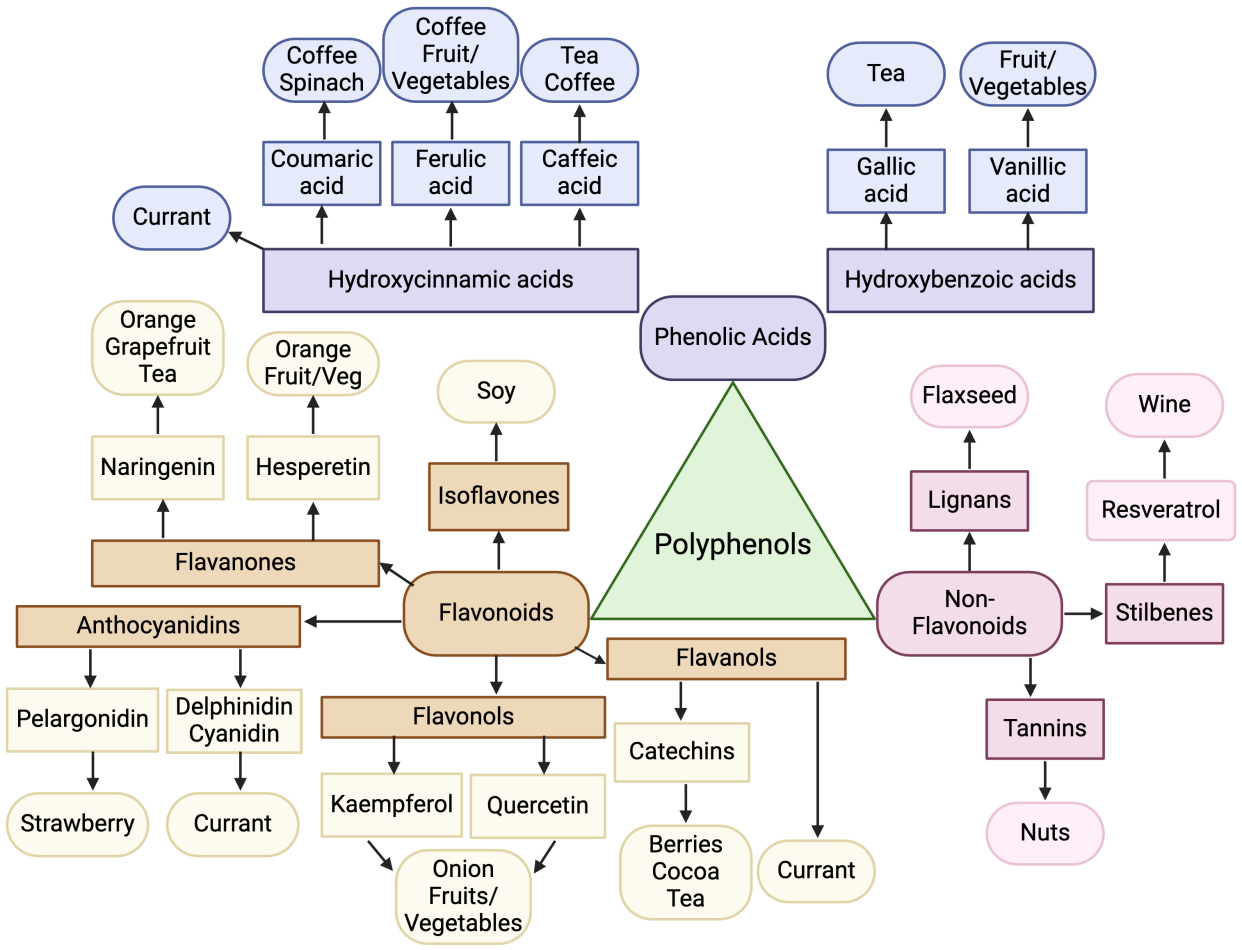
Map of polyphenol classes as biomarkers of dietary intake. Created with BioRender.com. Jackson, M. (2025) https://BioRender.com/f42q757.

In fact, one broad theme across food groupings was the majority of plant-based foods reviewed polyphenols and their subclasses as potential biomarkers. Figure 6 summarizes the connections between polyphenol classes and their respective plant-derived foods. For example, soy foods were unanimously represented by urinary isoflavones, a type of polyphenol of the sub-class of flavonoids; resveratrol as a biomarker for wine is a type of polyphenol under stilbenes; and naringenin and hesperetin, which fall under the polyphenol subgroup of flavanones, had strong representation as biomarkers of citrus fruit intake. Epicatechins were found to be biomarkers of cocoa and tea, which fall under flavanols, a sub-class of flavonoids, while coffee was represented by coumaric acid and ferulic acid, that can be grouped into hydroxycinnamic acids, a sub-class of phenolic acids.

While several studies point to high fruit and vegetable consumption being represented by polyphenols, additional common biomarkers across plant foods, included sulfur-containing compounds, such as glucosinolates, sulfoxides, and sulfides. These compounds are generally found in vegetables in the *Brassica* family (88), including cruciferous vegetables of broccoli, kale and cabbage, as well as foods in the *Allium* family (89), like garlic, leek and onion; thus it is logical they are confirmed to be present in urine for these foods. Additionally, whole grains did not have a polyphenol biomarker, but strong consensus demonstrated the appropriate biomarker for whole grains are alkylresorcinols. While not a polyphenol, alkylresorcinols do contain a lipophilic polyphenol structure, naturally synthesized by plants (90). In contrast, animal-based foods reviewed were not represented by polyphenols, but instead more commonly represented by amino acid-related metabolites, such as 1-MH and 3-MH, as derivatives of the essential amino acid histidine (91). Biomarkers for dairy similarly were derived from its major food component, galactose, only found in dairy foods (92). An additional point of interest is that combined modeling (using a profile of metabolites) was only investigated in a few studies, but led to stronger prediction capabilities (50, 63, 82) and warrants further exploration of combining several metabolites to create biomarker profiles of food intake. For example, McNamara et al. sought to develop a biomarker panel for fruit intake containing proline betaine, hippurate, and xylose and was able to distinguish intake based off three ranges grams of fruit intake, improving descriptive abilities compared to a dichotomous detection of yes/no metabolite present (93). The results from the present review can continue to guide the investigation of the most promising groupings of metabolites to improve predictions of food intake.

This study is strengthened by its wide inclusion criteria, allowing for cross-examination across different groups of food intake. Very few food groups delivered a consensus of one biomarker for a particular food and thus future studies may benefit from investigating if a multiple metabolite profile has a stronger relationship with food intake. While an array of study designs increased the ability to examine a diverse set of food groups, it also limits comparability across studies. Additionally, many studies had small cohorts, where 71% had less than 50 participants, and diversity was equally as sparse, being predominantly white. There is evidence to suggest that nutrient metabolism may differ by ethnic/racial genetic background, such as polymorphisms that increase choline requirements (94) or metabolism of polyunsaturated fatty acids (95). Thus, biomarkers of diet intake, especially when derived from downstream metabolite products, should be further studied in diverse cohorts. Lastly, further research should expand upon the predictive ability, including accuracy and reliability, of these identified metabolites in order to increase clinical and research utility of urinary biomarkers.

Current evidence on urinary biomarkers may have utility in describing intake of broad food groups, such as citrus fruits, cruciferous vegetables, whole grains, and soy foods, but may lack the ability to clearly distinguish individual foods. The ability to reliably use urine as a method of biomarker attainment for dietary intake has the potential to transform diet assessment methods, remove barriers to care, and improve the ability to personalize nutrition interventions. Importantly, urine collection can be done at home, compared to blood-derived nutrient assessment, allowing for an improvement in collection burden and compliance, while also expanding healthcare access to rural areas and those with transportation barriers. While these urinary metabolites have been researched broadly within general healthy populations, future research can verify these as biomarkers of diet intake in disease-state populations. The overall improvement of exposure assessment methodology is a key step toward strengthening research data validity and accurately measuring outcomes in chronic disease management.

## Data Availability

The original contributions presented in the study are included in the article/Supplementary material, further inquiries can be directed to the corresponding author.
