# Supplementary material for: Urinary metabolites as biomarkers of dietary intake: a systematic review
Source: Front Nutr. 2025 May 15;12:1596543. doi: 10.3389/fnut.2025.1596543 (PMC12119289; doi:10.3389/fnut.2025.1596543)
Supplement: Supplementary file 2 [file Data_Sheet_1.pdf]

Search Strategy

Research Topic: What are the urinary biomarkers that can be utilized for accurate assessment of dietary intake, including individual foods and food groups?

Inclusion criteria: 18+, English only, peer reviewed, publication date range: 2000-2022

Exclusion criteria: abstracts only, validation studies

Terms gleaned from benchmark articles:

Diet: “dietary intake” OR “food intake” OR “habitual food intake” OR “dietary exposure” OR “habitual consumption” OR “dietary pattern” OR “dietary patterns” OR “dietary metabotype” OR “nutrition phenotyping” OR “nutrition metabotype” OR “diet therapy” OR “diet therapies” OR food* OR diet* OR "food intake" OR "individual food" OR "food group" OR "food component" OR "dietary component" OR "dietary pattern" OR "dietary intake" OR "dietary profile" OR "dietary assessment" OR "dietary consumption" OR "dietary exposure" OR nutritypes OR "nutritional supplement" OR "western diet" OR "mediterranean diet" OR "healthy eating index" OR "alternative healthy eating index" OR "prudent diet pattern" OR "dietary inflammatory index"

Biomarker: biomarker* OR “nutritional biomarker” OR “nutritional biomarkers” OR “dietary biomarker” OR “food biomarker” OR “nutrient biomarker” OR “food/nutrient biomarker” OR “food/nutrient biomarkers” OR “dietary exposure biomarker” OR “metablomics analysis” OR metablomic* OR “metabolite fingerprinting” OR metablome OR metabolite* OR “metabolic phenotyping” OR “metabolite stability” OR “metablomic assessment” OR “metabolic profile”

Urinary terms: “urinary biomarker” OR “urinary biomarkers” OR “urinary metabolite” OR “urine sample” OR “urine collection” OR “urine sampling” OR “urine samples” OR urine OR urinary OR “urine composition” OR “urine profile” OR “urinary profiles” OR “urinary profiling” OR “urine collection” OR “spot urine” OR “urinary marker” OR “urine marker”

Results will exclude children so all strategies will NOT out child OR children OR infant OR baby OR babies OR toddler* OR preschooler* OR “school aged” OR “middle school” OR “high school” OR teen* OR preteen* OR tween* OR youth OR pediatric* OR paediatric*

Embase Search strategy:

(biomarker*:ab,ti OR 'nutritional biomarker':ab,ti OR 'nutritional biomarkers':ab,ti OR 'dietary biomarker':ab,ti OR 'food biomarker':ab,ti OR 'nutrient biomarker':ab,ti OR 'food/nutrient biomarker':ab,ti OR 'food/nutrient biomarkers':ab,ti OR 'dietary exposure biomarker':ab,ti OR 'metablomics analysis':ab,ti OR metablomic*:ab,ti OR 'or metabolite fingerprinting':ab,ti OR metablome:ab,ti OR metabolite*:ab,ti OR 'metabolic phenotyping':ab,ti OR 'metabolite stability':ab,ti OR 'metablomic assessment':ab,ti OR 'metabolic profile':ab,ti)

AND

('habitual food intake':ab,ti OR 'habitual consumption':ab,ti OR 'dietary pattern':ab,ti OR 'dietary patterns':ab,ti OR 'dietary metabotype':ab,ti OR 'nutrition phenotyping':ab,ti OR 'nutrition metabotype':ab,ti OR 'diet therapy':ab,ti OR 'diet therapies':ab,ti OR food*:ab,ti OR diet*:ab,ti OR 'food intake':ab,ti OR 'individual food':ab,ti OR 'food group':ab,ti OR 'food component':ab,ti OR 'dietary component':ab,ti OR 'dietary intake':ab,ti OR 'dietary profile':ab,ti OR 'dietary assessment':ab,ti OR 'dietary consumption':ab,ti OR 'dietary exposure':ab,ti OR nutritypes:ab,ti OR 'nutritional supplement':ab,ti OR 'western diet':ab,ti OR 'mediterranean diet':ab,ti OR 'healthy eating index':ab,ti OR 'alternative healthy eating index':ab,ti OR 'prudent diet pattern':ab,ti OR 'dietary inflammatory index':ab,ti)

AND

('urinary biomarker':ab,ti OR 'urinary biomarkers':ab,ti OR 'urinary metabolite':ab,ti OR 'urine sample':ab,ti OR 'urine sampling':ab,ti OR 'urine samples':ab,ti OR urine:ab,ti OR urinary:ab,ti OR 'urine composition':ab,ti OR 'urine profile':ab,ti OR 'urinary profiles':ab,ti OR 'urinary profiling':ab,ti OR 'urine collection':ab,ti OR 'spot urine':ab,ti OR 'urinary marker':ab,ti OR 'urine marker':ab,ti)

NOT

('child'/exp OR child OR 'children'/exp OR children OR infant* OR 'baby'/exp OR baby OR babies OR toddler* OR preschooler* OR 'school aged' OR 'middle school'/exp OR 'middle school' OR 'high school'/exp OR 'high school' OR teen* OR preteen* OR tween* OR 'youth'/exp OR youth OR pediatric* OR paediatric*)

PubMed search strategy:

((((("Eating"[Mesh]) OR "Nutritional Status"[Mesh]) OR ("dietary intake"[Title/Abstract] OR "food intake"[Title/Abstract] OR "habitual food intake"[Title/Abstract] OR "dietary exposure"[Title/Abstract] OR "habitual consumption"[Title/Abstract] OR "dietary pattern"[Title/Abstract] OR "dietary patterns"[Title/Abstract] OR "dietary metabotype"[Title/Abstract] OR "nutrition phenotyping"[Title/Abstract] OR "nutrition metabotype"[Title/Abstract] OR "diet therapy"[Title/Abstract] OR "diet therapies"[Title/Abstract] OR food*[Title/Abstract] OR diet*[Title/Abstract] OR "food intake"[Title/Abstract] OR "individual food"[Title/Abstract] OR "food group"[Title/Abstract] OR "food component"[Title/Abstract] OR "dietary component"[Title/Abstract] OR "dietary pattern"[Title/Abstract] OR "dietary intake"[Title/Abstract] OR "dietary profile"[Title/Abstract] OR "dietary assessment"[Title/Abstract] OR "dietary consumption"[Title/Abstract] OR "dietary exposure"[Title/Abstract] OR nutritypes[Title/Abstract] OR "nutritional supplement"[Title/Abstract] OR "western diet"[Title/Abstract] OR "mediterranean diet"[Title/Abstract] OR "healthy eating index"[Title/Abstract] OR "alternative healthy eating index"[Title/Abstract] OR "prudent diet pattern"[Title/Abstract] OR "dietary inflammatory index"[Title/Abstract])) AND (("biomarkers"[MeSH Terms] OR (biomarker*[Title/Abstract] OR "nutritional biomarker"[Title/Abstract] OR "nutritional biomarkers"[Title/Abstract] OR "dietary biomarker"[Title/Abstract] OR "food biomarker"[Title/Abstract] OR "nutrient

biomarker"[Title/Abstract] OR "food/nutrient biomarker"[Title/Abstract] OR "food/nutrient biomarkers"[Title/Abstract] OR "dietary exposure biomarker"[Title/Abstract] OR "metablomics analysis"[Title/Abstract] OR metablomic*[Title/Abstract] OR "metabolite fingerprinting"[Title/Abstract] OR metablome[Title/Abstract] OR metabolite*[Title/Abstract] OR "metabolic phenotyping"[Title/Abstract] OR "metabolite stability"[Title/Abstract] OR "metablomic assessment"[Title/Abstract] OR "metabolic profile"[Title/Abstract])) AND (("Urinalysis"[Mesh] OR "Urine"[Mesh]) OR ("urinary biomarker"[Title/Abstract] OR "urinary biomarkers"[Title/Abstract] OR "urinary metabolite"[Title/Abstract] OR "urine sample"[Title/Abstract] OR "urine collection"[Title/Abstract] OR "urine sampling"[Title/Abstract] OR "urine samples"[Title/Abstract] OR urine[Title/Abstract] OR urinary[Title/Abstract] OR "urine composition"[Title/Abstract] OR "urine profile"[Title/Abstract] OR "urinary profiles"[Title/Abstract] OR "urinary profiling"[Title/Abstract] OR "spot urine"[Title/Abstract] OR "urinary marker"[Title/Abstract] OR "urine marker"[Title/Abstract] OR))) NOT (child OR children OR infant* OR baby OR babies OR toddler* OR preschooler* OR "school aged" OR "middle school" OR "high school" OR teen* OR preteen* OR tween* OR youth OR pediatric* OR paediatric*)

CINAHL search strategy:

TI (“dietary intake” OR “food intake” OR “habitual food intake” OR “dietary exposure” OR “habitual consumption” OR “dietary pattern” OR “dietary patterns” OR “dietary metabotype” OR “nutrition phenotyping” OR “nutrition metabotype” OR “diet therapy” OR “diet therapies” OR food* OR diet* OR "food intake" OR "individual food" OR "food group" OR "food component" OR "dietary component" OR "dietary pattern" OR "dietary intake" OR "dietary profile" OR "dietary assessment" OR "dietary consumption" OR "dietary exposure" OR nutritypes OR "nutritional supplement" OR "western diet" OR "mediterranean diet" OR "healthy eating index" OR "alternative healthy eating index" OR "prudent diet pattern" OR "dietary inflammatory index") OR AB (“dietary intake” OR “food intake” OR “habitual food intake” OR “dietary exposure” OR “habitual consumption” OR “dietary pattern” OR “dietary patterns” OR “dietary metabotype” OR “nutrition phenotyping” OR “nutrition metabotype” OR “diet therapy” OR “diet therapies” OR food* OR diet* OR "food intake" OR "individual food" OR "food group" OR "food component" OR "dietary component" OR "dietary pattern" OR "dietary intake" OR "dietary profile" OR "dietary assessment" OR "dietary consumption" OR "dietary exposure" OR nutritypes OR "nutritional supplement" OR "western diet" OR "mediterranean diet" OR "healthy eating index" OR "alternative healthy eating index" OR "prudent diet pattern" OR "dietary inflammatory index") OR (MH "Nutritional Status") OR (MH "Nutrition+") OR (MH "Diet+")

AND

TI (biomarker* OR “nutritional biomarker” OR “nutritional biomarkers” OR “dietary biomarker” OR “food biomarker” OR “nutrient biomarker” OR “food/nutrient biomarker” OR “food/nutrient biomarkers” OR “dietary exposure biomarker” OR “metablomics analysis” OR metablomic* OR “metabolite fingerprinting” OR metablome OR metabolite* OR “metabolic phenotyping” OR “metabolite stability” OR “metablomic assessment” OR “metabolic profile”) OR AB (biomarker* OR “nutritional biomarker” OR “nutritional biomarkers” OR “dietary biomarker” OR “food biomarker” OR “nutrient biomarker” OR “food/nutrient biomarker” OR “food/nutrient biomarkers” OR “dietary exposure biomarker” OR “metablomics analysis” OR metablomic* OR “metabolite

fingerprinting” OR metablome OR metabolite* OR “metabolic phenotyping” OR “metabolite stability” OR “metablomic assessment” OR “metabolic profile”) OR (MH "Metabolites")

AND

TI (“urinary biomarker” OR “urinary biomarkers” OR “urinary metabolite” OR “urine sample” OR “urine collection” OR “urine sampling” OR “urine samples” OR urine OR urinary OR “urine composition” OR “urine profile” OR “urinary profiles” OR “urinary profiling” OR “urine collection” OR “spot urine” OR “urinary marker” OR “urine marker”) OR AB (“urinary biomarker” OR “urinary biomarkers” OR “urinary metabolite” OR “urine sample” OR “urine collection” OR “urine sampling” OR “urine samples” OR urine OR urinary OR “urine composition” OR “urine profile” OR “urinary profiles” OR “urinary profiling” OR “urine collection” OR “spot urine” OR “urinary marker” OR “urine marker”) OR (MH "Urinalysis") OR (MH "Urine")

NOT

(child OR children OR infant* OR baby OR babies OR toddler* OR preschooler* OR “school aged” OR “middle school” OR “high school” OR teen* OR preteen* OR tween* OR youth OR pediatric* OR paediatric*)
